# Efficacy of Altered Two-Point Fixation in Zygomaticomaxillary Complex Fracture

**DOI:** 10.1155/2020/8537345

**Published:** 2020-03-18

**Authors:** Jun Hyeok Kim, Ye Sol Kim, Deuk Young Oh, Young Joon Jun, Jong Won Rhie, Suk-Ho Moon

**Affiliations:** Department of Plastic & Reconstructive Surgery, College of Medicine, The Catholic University of Korea, Seoul, Republic of Korea

## Abstract

**Purpose:**

To reconstruct a zygomaticomaxillary complex (ZMC) fracture, zygomaticofrontal (ZF) suture is the most reliable site to assess anatomical alignment and to secure rigidity. It has been chosen primary site to be fixed, but approach through the lateral eyebrow incision may leave a visible scar. This study suggests altered two-point fixation of ZMC fracture without accessing the ZF suture.

**Methods:**

In the retrospective study, a total of 40 patients with ZMC fracture were divided into two groups (group 1, two-point fixation and group 2, three-point fixation). Patient demographics and follow-up were evaluated, and degree of reduction including cortical gaps of ZF and inferior orbital (IO) area, protruding difference of zygoma, and malar difference using asymmetry index were measured through preoperative and postoperative CT.

**Results:**

Preoperatively, the means of ZF displacement, IO displacement, protruding difference of zygoma, and facial asymmetry index between the groups were not statistically different. The result was the same after the operation. However, all variables were significantly different before and after surgery within each group. Moreover, mean operation time was significantly different between groups (*P* value = 0.026).

**Conclusion:**

Altered two-point fixation in ZMC fracture excluding incision approaching the ZF provides surgical efficacy and similar surgical outcomes to three-point fixation but offers reduced operation time and fewer complications.

## 1. Introduction

Facial appearance affects the foundation of an individual's personality, and facial change due to injury can cause harmful alteration in one's sense of self and how one interacts and expresses oneself in society [1]. The zygomatic bone is the most prominent and characteristic in the midface [2], and its traumatic fracture may lead to crucial deformity of the face [3, 4]. It is essential to restore the bony structure of the zygoma to its original shape.

The standard treatment for zygomaticomaxillary complex (ZMC) fracture has been open reduction and internal fixation (ORIF) [5], and sites of one-, two-, or three-point fixation are selected based on stability of the fractured zygoma [6, 7]. Among fixation sites, the zygomaticofrontal (ZF) suture followed by zygomaticosphenoidal (ZS) suture has been the single most reliable site for anatomical alignment and secure fixation [8, 9]. Thus, this site has been the primary location of fixation [2, 5, 6, 10–13] even in 1-point fixation [14, 15]. However, the ORIF approach of ZF suture through a lateral eyebrow incision may leave visible scars, uncomfortable palpability of plates on thin skin, and risk of drill penetration into the anterior cranial fossa [8, 12, 16].

Thus, this study compares the results of altered two-point fixation at the zygomaticomaxillary (ZM) buttress and infraorbital rim (IO) with three-point fixation with a ZF suture in ZMC fracture.

## 2. Patients and Methods

In this single-center, retrospective study, we evaluated the medical records and three-dimensional (3D) computed tomography (CT) scans of 117 patients with type B of ZMC fracture [17] between December 2015 and April 2019.

The inclusion criteria were as follows:

1. Diagnosis of unilateral ZMC tetrapod fracture with preoperative radiological evaluation including 3D CT
2. ORIF within two weeks after injury
3. Postoperative evaluation including clinical outcomes and radiological examination including 3D CT within three months postoperative.

The exclusion criteria were as follows:

1. Only fracture of the zygomatic arch (case of closed reduction, type A injuries) [17]
2. Complex or combined fracture needing ORIF such as fracture of the mandible or frontal bone as well as type C injuries [17]
3. No preoperative or postoperative 3D CT scans
4. No postoperative evaluation (because the patient did not visit the outpatient clinic of the Department of Plastic and Reconstructive Surgery of our medical institution around three months postoperatively).

The present study was approved by the institutional review board (IRB) of our medical institution (IRB approval number: KC19RESI0427). The requirement for informed consent was waived due to the retrospective nature of the study.

A total of 40 patients was included in this study and divided into two groups. Group 1 was composed of 20 patients who underwent two-point (ZM buttress and IO area) ORIF through buccogingival and subciliary incisions, and group 2 comprised 20 patients who underwent three-point (ZM buttress, IO, and ZF areas) ORIF through buccogingival, subciliary, and lateral eyebrow incisions.

### 2.1. Operative Technique

The fracture sites were exposed including the fracture line so that plates could be applied under general anesthesia. The ZM area was exposed approximately 1.0 cm from the infraorbital rim for insertion of 6 mm dual-top screws (Jeil Medical Corporation, Seoul, Republic of Korea) perpendicular to the direction of reduction. The screw was inserted, and a 26-gauge wire was passed through a hole in the screw head. Displaced fragments were reduced anatomically by retracting the wire. If a bone fragment including the zygomatic arch was insufficiently reduced, another screw was inserted on the ZM buttress, and the two screws were retracted for anatomical reduction. In this process, the indicators of accurate reduction were concave alignment of the orbital floor, straightened continuity of the infraorbital rim, and palpation of the lateral rim for group 1 and alignment of the greater wing of the ZS for group 2. After confirming anatomical reduction, each fracture site was fixed with absorbable plates and screws (Inion CPS, Tampere, Finland). The wound was thoroughly irrigated, and hemostasis was confirmed. The periosteum, skin, and mucosa were closed in a layer-by-layer manner.

### 2.2. Management

Patients consumed a liquid diet for three days after surgery. They could then eat a general diet composed of soft food for six months. After two months, 3D CT scan was performed to confirm correct alignment and maintenance of bone fragments.

### 2.3. Measurement

**Figure 1:**
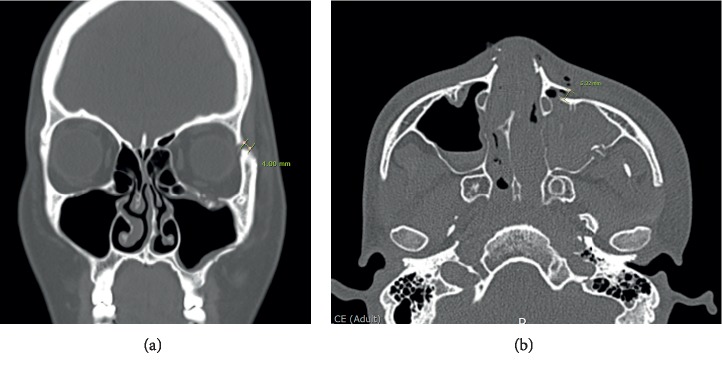
Degree of reduction. Gauging displacement distance between outer cortical bones. (a) Measurement of the cortical gap of the zygomaticofrontal suture. (b) Measurement of the cortical gap of the inferior orbital rim.

**Figure 2:**
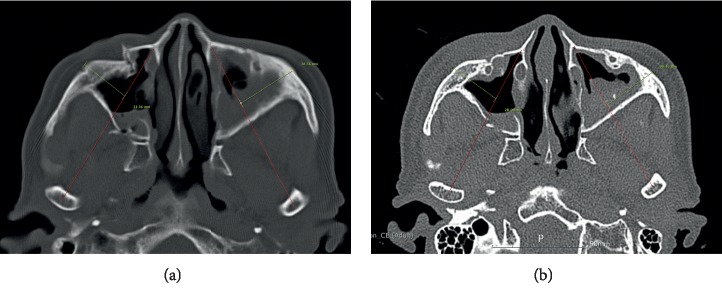
The protruding difference of zygoma: compared by measuring the distance from the most prominent point of each zygomatic arch to A line (A line: a virtual line from the pyriform aperture to the condyle of the mandible). (a) Preoperative measurement. (b) Postoperative measurement.

**Figure 3:**
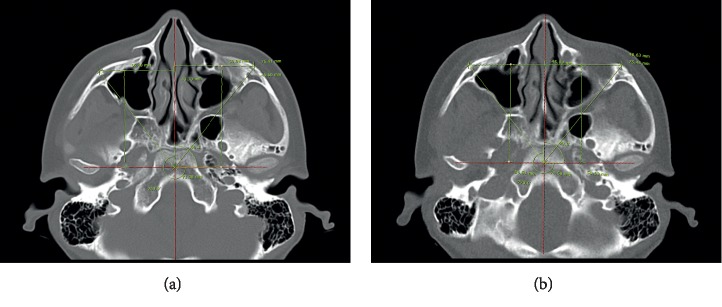
The asymmetry index of zygomatic prominence: comparing the difference in the two prominences of the zygomatic bone. (a) Preoperative measurement. (b) Postoperative measurement (Hr: right horizontal length, Hl: left horizontal length, Vr: right vertical length, Vl: left vertical length, Dr: right distance from midpoint, and Dl: left distance from midpoint). Asymmetry index = $\sqrt{{\left(\text{Hr}-\text{Hl}\right)}^{2}+{\left(\text{Vr}-\text{Vl}\right)}^{2}+{\left(\text{Dr}-\text{Dl}\right)}^{2}}$.

Patient demographics and follow-up data were evaluated, and the degree of reduction including cortical gaps of ZF and IO area (Figure 1), protruding difference of zygoma, and malar difference using asymmetry index were measured via preoperative and postoperative 3D CT. The protruding difference of zygoma was compared by measuring the distance from the most prominent point of each zygomatic arch to A line. (A line: a virtual line from the pyriform aperture to the condyle of the mandible) (Figure 2). The asymmetry index was calculated using the following formula to compare the difference in prominence of the zygomatic arches [18, 19] (Figure 3):

(1) $$\begin{array}{c} \text{Asymmetry index}=\sqrt{{\left(\text{Hr}-\text{Hl}\right)}^{2}+{\left(\text{Vr}-\text{Vl}\right)}^{2}+{\left(\text{Dr}-\text{Dl}\right)}^{2}}, \end{array}$$

where Hr is right horizontal length, Hl is left horizontal length, Vr is right vertical length, Vl is left vertical length, Dr is right distance from midpoint, and Dl is left distance from midpoint.

### 2.4. Statistical Analysis

For nominal variables, fractions in percentages were calculated, and Fisher's exact test was used for comparison. For continuous variables, the mean and SD were used for description, and the difference between groups was compared using Mann–Whitney test or paired *T* test. *P* value less than 0.05 indicated a statistically significant difference.

## 3. Results

The baseline characteristics and demographic data of the patients are summarized in Table 1. The groups had no differences in age, sex, lesion side, causes of trauma, concomitant injuries, and operation delay. Preoperatively, the means of ZF displacement, IO displacement, protruding difference of zygoma, and facial asymmetry index of group 1 were 2.15 ± 1.48, 4.07 ± 2.22, 3.50 ± 2.94, and 5.82 ± 2.42, respectively, and those of group 2 were 2.47 ± 2.26, 5.24 ± 3.55, 2.50 ± 1.39, and 4.84 ± 2.21. No variable was statistically different between groups (Table 2). On the other hand, all variables were significantly different before and after surgery within each group (Table 3). Moreover, the means of operation time, hospital stay, and follow-up period of group 1 were 96.25 ± 26.07, 6.00 ± 1.59, and 63.35 ± 35.54, respectively, and those of group 2 were 116.02 ± 28.50, 5.45 ± 1.00, and 93.50 ± 121.20. Operation time was significantly different between groups (*P* value: 0.026), but average duration of hospital stay and follow-up period were similar (*P* values: 0.338 and 0.763, respectively) (Table 4).

## 4. Discussion

The results showed that preoperative and postoperative variables of the two groups were not statistically different, while the variables before and after surgery within each group were significantly changed. In other words, the surgical results of the two groups were the same, despite one fewer incision used to access the ZF of group 1. The altered two-point fixation, excluding an incision approaching the ZF, required a shorter operation time, allowing a more efficient surgery than three-point fixation of ZMC fracture.

The primary goal of this study is to avoid lateral brow incision and ORIF of the ZF suture to achieve reconstruction of ZMC fracture without sequelae of unnecessary scar, palpation of plates, and ectropion [8, 12, 16]. In addition, the operation time is shortened. Instead, the standard of anatomical alignment is based on the surface of the orbital floor and the continuity of IO rim in the transverse direction and the ZM complex as the basis of the vertical buttress. Although transconjunctival incision at the upper eyelid has been tried to avoid transcutaneous incisions for ZF sutures [20], it has not been generally accepted. One study attempted to select ORIF sites other than the ZF suture [16], but it focused on patient satisfaction without quantitative analysis. In the present study, statistical analysis and a follow-up investigation of the results are clearly presented.

In the traditional three-point ORIF, ZF, IO, and ZM have been essential points for achieving stability in a ZMC fracture [2, 15, 21] and have been approached through lateral brow, subciliary or transconjunctival, and intraoral incisions, respectively. However, approaches via three points require a long surgery time and may result in complications including ectropion and noticeable scars [22]. Although selection of ORIF with fewer than three points has been studied, most techniques include the ZF suture [2, 5, 6, 9–15, 22].

The zygomatic bone occupies the most prominent of malar eminences, and it forms the facial width and a major buttress of the midface [2]. It has a tetrapod structure composed of 4 articulations, referred to as the ZM, ZF, zygomaticotemporal (ZT), and ZS sutures [7, 23]. ZMC fracture, including all tetrapod, is the second most common facial bone fracture [7, 24] and may result in critical deformity [3, 4]. Accuracy of ZMC reconstruction is essential to restore orbital volume and to reestablish facial projection and width [25].

The classification of the present study distinguishes three types of ZMC fractures: A, B, and C [17]. According to this classification, type A injury means the fracture of isolated one component of the buttress, such as the zygomatic arch (type A1), the lateral orbital wall (type A2), and the inferior orbital rim (type A3). Type B fracture includes all four buttresses, so-called tetrapod fracture which was the indication of the surgery in the present study. Type C injury is classified as complex fractures with comminution of the zygomatic bone. The indication of the ORIF in the present study was only type B ZMC fracture.

Fracture healing is the process in which bony tissue restores its innate physical and mechanical properties [26]. In the beginning 4 to 6 weeks of bone healing, the callus is frail, and mechanical stability is a crucial factor to form an appropriate callus by means of external or internal fixation. This results in gradual maturation of the callus from woven to lamellar bone [27]. If fixation is not successful, the callus may not be calcified, and an unstable fibrous union may be generated [26]. Therefore, rigid or semirigid fixation of fractured bone is essential, and the fixation by absorbable plates and screw provides good long-term stability to achieve the healing of the ZMC fracture [28–33].

The standard treatment of ZMC fracture is ORIF [5]; except for the ZT suture, the locations and number of fixation points remain in dispute [6, 16]. Because isolated fractures of the ZT complex or zygomatic arch are often mild [1, 6], closed reduction is the effective treatment through the Gillies approach or Keen's approach [6, 34]. Gillies incision is no more than 2.5 cm parallel to the hair follicles through the temporal scalp within the hairline, and the Dingman elevator passes between the deep fascia and the temporalis muscle [1, 23].

The ZF complex is a narrow and dense bony region that acts as the lateral vertical maxillary buttress and is a reliable site to secure stability [9] and to evaluate well-reduced alignment without rotational deformity of the ZS suture [8]. The ZF complex is thus most commonly selected for fixation [2, 5, 6, 10–15]. A Dingman elevator can be inserted under the zygomatic arch for effective reduction through upper eyelid, lateral brow, and extended lower eyelid incisions [35–38]. However, ORIF of the ZF suture accompanying these incisions often produces sequelae, including a striking scar, unpleasant perception of plates via thin skin, ectropion, and risk of injury to the anterior cranial fossa [8, 12, 16].

The IO has a role in the upper transverse maxilla with the ZM complex across the ZT suture [8, 23], and it can be accessed through numerous incisions including subciliary, subtarsal, intraorbital, and transconjunctival [5, 23, 39]. In cases of diplopia, enophthalmos, and comminuted fracture, the inferior orbital rim can be explored simultaneously to evaluate accompanying impure blow-out fracture [40]. Furthermore, this study suggests that exploring the inferior orbital wall and alignment of the IO can be indicators of successful anatomical alignment. In group 2, in the immediate postoperative reduction state and postoperative radiological test, evaluating the concave surface of the orbital floor and the straightened continuity of the IO was a reliable, acceptable standard.

The ZM and pterygomaxillary complexes are the major vertical buttresses for mastication [10] and unite the maxillary alveolus with the ZT complex. Keen's approach is an intraoral route using a mucosal incision [23] to expose ZM and pterygomaxillary buttresses [1, 41, 42]. This approach can be used in closed reduction through the stab incision.

Lateral brow incision permits a Dingman approach, which provides the strongest rotary force on the ZMC fracture segment from the caudal direction in the vertical axis. The present study used only Keen's approach, involving a dual-top screw for restoration in severe displacement or impaction. Dual-top screws are useful especially in cases where the fractured fragment is displaced downward and rotated inward without excessive reduction force [41, 43]. As it is unnecessary to expose the buttress widely or to separate zygomatic segments from soft tissue and muscle in this procedure, the rate of soft tissue complications including cheek drooping is low.

Other methods to overcome the inadequate vector for reduction of the zygoma are Kirschner's wire [44, 45] and T-bar screw [15, 46] traction. Both methods allow precise three-dimensional manipulation of a fractured segment, after which reduction is controllable in any vector and direction can involve an elevator via Keen's approach.

The limitations of the present study are that it is not a randomized controlled trial, but the retrospective study. And the exact measurement point of zygomatic redirection cannot be defined. Also, the number of participating patients was small.

## 5. Conclusion

Altered two-point fixation of zygomaticomaxillary complex fracture, excluding an incision approaching the ZF, provides surgical efficacy. The method presents the same surgical outcomes as traditional three-point fixation. However, because altered two-point fixation includes one less incision, it requires less operation time and reduces the noticeable scar and complications of a palpable and exposed plate via the skin.

## Figures and Tables

**Table 1:** Patient characteristics and demographic data.

	Group 1	Group 2	*P* value
Age, year	45.40 ± 21.36	53.10 ± 20.57	0.253
Sex			0.354
Male	13 (65%)	14 (70%)	
Female	7 (35%)	6 (30%)	
Lesion side			0.642
Right	6 (30%)	8 (40%)	
Left	14 (70%)	12 (60%)	
Cause			0.510
Traffic accident	2 (10%)	4 (20%)	
Fall down	9 (45%)	11 (55%)	
Assault	3 (15%)	2 (10%)	
Accidental bump	5 (25%)	3 (15%)	
Concomitant injuries	3 (15%)	4 (20%)	0.509
Operation delay, day	9.60 ± 3.65	10.10 ± 4.24	0.692

**Table 2:** Surgical outcomes: comparison of group 1 and group 2.

	Group 1	Group 2	*P* value
Preoperative variables (mm)			
ZF displacement	2.15 ± 1.48	2.47 ± 2.26	0.603
IO displacement	4.07 ± 2.22	5.24 ± 3.55	0.383
Protruding difference of zygoma	3.50 ± 2.94	2.50 ± 1.39	0.395
Asymmetry index	5.82 ± 2.42	4.84 ± 2.21	0.189
Preoperative variables (mm)			
ZF displacement	1.25 ± 1.13	1.48 ± 1.24	0.556
IO displacement	1.55 ± 1.55	0.85 ± 1.24	0.136
Protruding difference of zygoma	1.53 ± 1.60	1.64 ± 1.11	0.324
Asymmetry index	2.35 ± 0.85	2.43 ± 0.85	0.759

**Table 3:** Surgical outcomes: comparison of preoperative and postoperative variables within each group.

	Preoperative	Postoperative	*P* value
Variable of group 1 (mm)			
ZF displacement	2.15 ± 1.48	1.25 ± 1.13	0.006^*∗*^
IO displacement	4.07 ± 2.22	1.55 ± 1.55	<0.001^*∗∗∗*^
Protruding difference of zygoma	3.50 ± 2.94	1.53 ± 1.60	0.012^*∗*^
Asymmetry index	5.82 ± 2.42	2.35 ± 0.85	<0.0001^*∗∗∗∗*^
Variable of group 2 (mm)			
ZF displacement	2.47 ± 2.26	1.48 ± 1.24	0.022^*∗*^
IO displacement	5.24 ± 3.55	0.85 ± 1.24	<0.0001^*∗∗∗∗*^
Protruding difference of zygoma	2.50 ± 1.39	1.64 ± 1.11	0.024^*∗*^
Asymmetry index	4.84 ± 2.21	2.43 ± 0.85	<0.0001^*∗∗∗∗*^

**Table 4:** Operation time, hospitalization, and follow-up period.

	Group 1	Group 2	*P* value
Operation time, minute	96.25 ± 26.07	116.02 ± 28.50	0.026^*∗*^
Hospital stay, day	6.00 ± 1.59	5.45 ± 1.00	0.338
Follow-up period, day	63.35 ± 35.54	93.50 ± 121.20	0.763

## Data Availability

The data from CT measurement used to support the findings of this study are available from the corresponding author upon request.
